# New Fuels for a Failing Engine: The Impact of Novel Heart Failure Drugs on Functional Capacity

**DOI:** 10.31083/RCM41919

**Published:** 2025-09-25

**Authors:** Nikita Baracchini, Teresa Maria Capovilla, Simona Costantino, Fiorella Puttini, Elisabetta Salvioni, Irene Mattavelli, Massimo Valenti, Emilia d'Elia, Elena Bertarelli, Piergiuseppe Agostoni, Gianfranco Sinagra, Massimo Mapelli

**Affiliations:** ^1^Cardiothoracovascular Department, Azienda Sanitaria Universitaria Giuliano-Isontina (ASUGI), University of Trieste, 34149 Trieste, Italy; ^2^Postgraduate School of Cardiovascular Medicine University of Trieste, Azienda Sanitaria Universitaria Giuliano-Isontina (ASUGI), University of Trieste, 34149 Trieste, Italy; ^3^Centro Cardiologico Monzino, IRCCS, 20138 Milan, Italy; ^4^Department of Clinical Sciences and Community Health, Cardiovascular Section, University of Milan, 20122 Milan, Italy; ^5^Cardiovascular Department, ASST Papa Giovanni XXIII, 24127 Bergamo, Italy

**Keywords:** cardiopulmonary exercise testing, heart failure, novel drugs, functional capacity

## Abstract

Functional impairment is a hallmark of heart failure (HF) and a strong prognostic factor. Cardiopulmonary exercise testing (CPET) provides a robust and objective assessment of exercise capacity; however, the impact of new pharmacotherapies on CPET parameters remains largely uncharacterized systematically. This review examines the influence of contemporary HF therapies on functional capacity, with particular focus on CPET-derived metrics, such as peak oxygen uptake (VO_2_ peak), ventilatory efficiency (VE/VCO_2_ slope), and oxygen uptake efficiency slope (OUES). A critical synthesis of randomized trials, observational studies, and meta-analyses was performed to assess the effects of both conventional (angiotensin-converting enzyme (ACE) inhibitors, beta-blockers, mineralocorticoid receptor antagonists (MRAs)) and novel agents (angiotensin receptor neprilysin inhibitor (ARNIs), sodium–glucose cotransporter-2 (SGLT2) inhibitors, glucagon-like peptide-1 (GLP)-1 receptor agonists, vericiguat, finerenone) on CPET outcomes. Conventional therapies provide modest improvements in CPET indices, whereas sacubitril/valsartan and SGLT2 inhibitors show more consistent and clinically meaningful benefits across different HF phenotypes. Vericiguat provided preliminary promise in improving VO_2_ peak and ventilatory parameters. Meanwhile, evidence for GLP-1 receptor agonists and finerenone remains limited or inconclusive. Heterogeneity across studies, in terms of the timing of CPET follow-up and baseline functional status, emerged as important modulators of the observed outcomes. Novel HF therapies can potentially improve exercise capacity beyond symptomatic relief, supporting a shift toward CPET-based endpoints in HF clinical trials. Personalized CPET monitoring may optimize therapeutic strategies and better reflect meaningful functional gains in HF populations.

## 1. Introduction

Heart failure (HF) is a complex clinical syndrome characterized by the heart’s inability to meet the body’s metabolic demands. It affects over 64 million people globally and is associated with substantial mortality, morbidity, and healthcare expenditure [1, 2]. HF is the leading cause of hospitalization among individuals over 65 years of age [1]. A defining feature of HF is reduced functional capacity, often manifesting as exertional dyspnea, fatigue, and limited exercise tolerance. These symptoms are strong predictors of adverse outcomes, including recurrent hospitalization and mortality [2].

The assessment of functional capacity in HF patients extends beyond mere symptom evaluation, offering a quantitative measure of their ability to perform physical tasks. Cardiopulmonary exercise testing (CPET) provides a comprehensive assessment of the integrated responses of the cardiovascular, respiratory, and muscular systems to exercise. Key parameters such as peak oxygen consumption (VO_2_ peak), ventilatory efficiency (VE/VCO_2_ slope), and oxygen uptake efficiency slope (OUES) offer valuable insights into disease severity and prognosis [3]. Specifically, peak oxygen uptake (VO_2_ peak), defined as the maximum rate of VO_2_ during exercise, serves as a powerful predictor of mortality and morbidity in HF patients. Furthermore, the VE/VCO_2_ slope, reflecting ventilatory efficiency, contributes to risk stratification and identification of patients at higher risk of adverse outcomes [4, 5, 6].

**Fig. 1.**
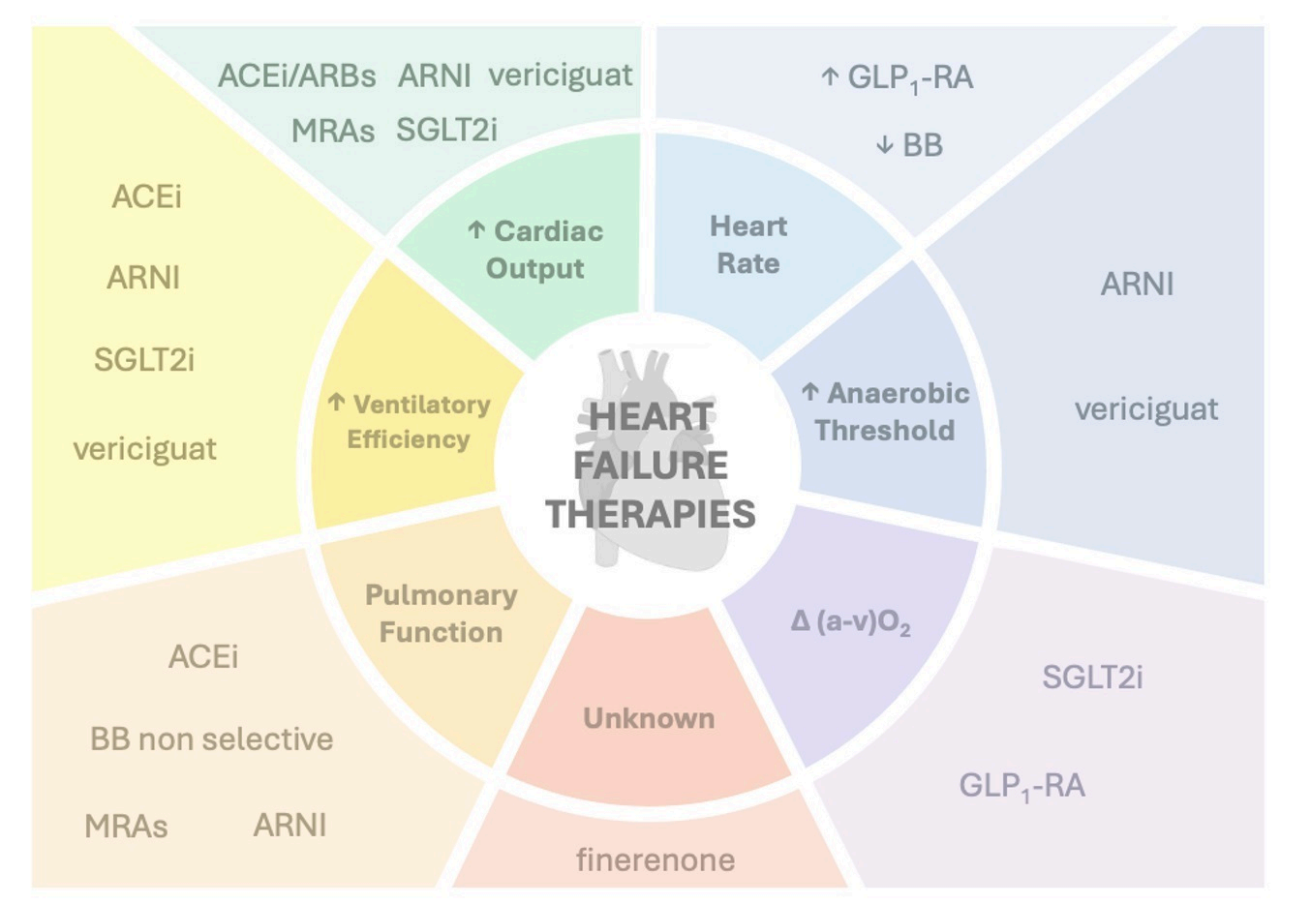
**Simplified mechanism of actions of heart failure therapies on CPET performance**.Primary targets and downstream effects of key pharmacological agents used in heart failure, with emphasis on their influence on exercise physiology as assessed by CPET. Heart failure therapies act through distinct mechanisms to modulate VO_2_ peak, ventilatory efficiency (VE/VCO_2_ slope), oxygen pulse, and other CPET-derived metrics. Abbreviations: ACEi, Angiotensin-Converting Enzyme Inhibitors; ARNI, Angiotensin Receptor–Neprilysin Inhibitor; BB, Beta-Blockers; CPET, Cardiopulmonary Exercise Testing; GLP1-RA, Glucagon-Like Peptide-1 Receptor Agonists; MRAs, Mineralocorticoid Receptor Antagonists; SGLT2i, Sodium-Glucose Cotransporter 2 Inhibitors; (a–v)O_2_ , Arteriovenous Oxygen Difference; CO, Cardiac Output; HR, Heart Rate.

In recent years, the therapeutic landscape of HF has been transformed by the advent of novel pharmacological agents. Although traditional therapies—such as Angiotensin-converting enzyme inhibitors (ACE-i), beta-blockers, and mineralocorticoid receptor antagonists (MRAs)—effectively improve symptoms and reduce mortality, their impact on functional capacity, as measured by CPET, has been controversial and mostly modest. The emergence of new therapeutic strategies, including sacubitril/valsartan, sodium-glucose cotransporter-2 inhibitors (SGLT2i), vericiguat, and finerenone, has outlined new avenues for enhancing functional capacity in HF patients. Evidence suggests that these novel agents, acting through distinct mechanisms of action, exhibit the ability to improve CPET parameters and exercise tolerance, potentially translating into significant clinical benefits. Fig. 1 summarizes the effectes of HF therapies on exercise parameters.

This review aims to explore the impact of novel HF therapies on functional capacity, with a specific focus on CPET parameters. This manuscript provides insights and proposes potential pathophysiological mechanisms. It does not offer recommendations for or against the use of any specific drug, as such guidance requires evidence from randomized controlled trials.

Drugs related to specific cardiomyopathies, such as hypertrophic cardiomyopathy or cardiac amyloidosis, were deliberately excluded from this work, as they warrant a separate, dedicated analysis.

## 2. Methods

A critical synthesis of current literature was performed to assess the effects of both conventional (ACE inhibitors, beta-blockers, MRAs) and novel agents (ARNIs, SGLT2 inhibitors, GLP-1 receptor agonists, vericiguat, finerenone) on CPET outcomes.

The initial screening of articles was conducted using search engines such as PubMed and Scopus. The search keywords were “CPET”, “functional capacity”, “6MWT” and “heart failure”. Studies considered for inclusion primarily consisted of randomized trials, meta-analyses, observational studies, and case series. Case reports, outdated studies, non-English articles, and studies deemed irrelevant—i.e., those not evaluating the impact of the drug on functional capacity parameters—were excluded.

## 3. Traditional Therapies and CPET: A Foundation for Improvement

Conventional pharmacologic management of HF, encompassing ACE-i and Angiotensin II Receptor Blockers (ARBs), beta-blockers, and MRAs, are four pillars of therapy for patients with HFrEF [7, 8]. While the cardiovascular effects of these therapies have been extensively documented, their impact on integrated cardiopulmonary function, both at rest and during exercise, remains less explored.

### 3.1 ACE Inhibitors and Angiotensin II Receptor Blockers

ACE-i and ARBs have demonstrated a substantial impact on functional capacity and CPET parameters in patients with HF. Treatment with enalapril has been shown to increase exercise capacity by improving lung diffusion capacity (DLCO), exercise ventilatory efficiency (VE/VCO_2_ slope), and VO_2_ peak [9, 10]. In a randomized crossover trial, Guazzi *et al*. [11] reported that enalapril increased VO_2_ peak from 13.2 ± 2.0 to 15.3 ± 3.0 mL/Kg/min (*p* < 0.01), alongside significant reductions in VE/VCO_2_ slope and dead space (lower Vd/Vt radio), reflecting enhanced alveolar-capillary gas exchange. Furthermore, enalapril improved alveolar membrane conductance without significantly altering pulmonary capillary blood volume, suggesting that the benefits are primarily due to enhanced molecular diffusion across the alveolar-capillary membrane rather than hemodynamic changes​ [11]. Notably, these positive effects were blunted when enalapril was co-administered with Aspirin, highlighting a probable mechanism involving increased prostaglandin availability​ [9, 10]. Losartan improves exercise capacity in HF patients by enhancing peripheral muscle perfusion rather than altering pulmonary function. In randomised trials, treatment with losartan led to significant increases in VO_2_ peak, without affecting lung diffusion or ventilatory efficiency, suggesting a distinct mechanism compared to ACE-i [9, 10].

### 3.2 Beta-Blockers

Beta-blockers (BB) represent further foundational drugs for the management of HF. While their role in improving left ventricular function and reducing mortality is unequivocal, their impact on functional capacity remains complex and somewhat paradoxical. Although some studies have reported improvements, others have failed to demonstrate significant benefits [12, 13].

According to Fick’s law, exercise performance depends on several factors, including cardiac output and O_2_ extraction by muscles: VO_2_ peak = stroke volume (SV) × heart rate (HR) peak × arteriovenous O_2_ difference [14]. In this context, BB may theoretically influence all three key components of the Fick equation: they reduce peak HR, may modulate SV through improved diastolic filling and reverse remodeling, and could affect peripheral oxygen extraction. In particular, Montero and Flammer [15] emphasize that although BB significantly limit HR peak, potentially reducing convective O_2_ delivery, VO_2_ peak is often preserved, suggesting compensatory mechanisms such as increased arteriovenous oxygen difference. This implies that peripheral adaptations might buffer central limitations imposed by BB therapy, although these adjustments are heterogeneous across patient populations and BB types [15]. Meta-analyses have consistently demonstrated that BB do not significantly increase VO_2_ peak when compared to placebo (standardized mean difference [SMD] for VO_2_ peak, –0.04; 95% CI: –0.20–0.12; *p* = 0.61)​ [15]. However, their influence on functional status is more favorable, with significant improvements in New York Heart Association (NYHA) class and prolongation of exercise time by a mean of 44 seconds [15, 16].

Other investigations into the relationship between β-blocker therapy and exercise capacity have focused on pulmonary function. Nonselective β-blockers, such as carvedilol, have been observed to enhance ventilatory efficiency, yet simultaneously impair lung diffusion. Conversely, β_1_-selective agents, such as bisoprolol and nebivolol, demonstrate a more neutral effect on both ventilatory efficiency and pulmonary diffusion [9]. These findings suggest that the choice of β-blocker in HF should be individualized, considering the patient’s ventilatory profile and pulmonary diffusion capacity, particularly in those with impaired lung function or increased ventilatory response to exercise [9].

Finally, prescribing decisions should be guided by evidence-based indications with proven prognostic value to avoid potentially harmful or uncertain effects. This is the case in the study by Palau *et al*. [17], which showed an improvement in VO_2_ peak after the withdrawal of beta-blockers in patients with HFpEF and chronotropic incompetence.

### 3.3 Mineralocorticoid Receptor Antagonists

MRAs, including Spironolactone and Eplerenone, have significant benefits in reducing mortality and hospitalizations in patients with heart failure with reduced ejection fraction (HFrEF)​ [18, 19].

Many studies evaluated the cardioprotective and antifibrotic effects on the lungs of Spironolactone and Eplerenone [20]. Furthermore, enhancing the endothelin pathway and the derived Nitric Oxide (NO), MRAs prevent or reverse pulmonary vascular remodeling and pulmonary artery hypertension [21]. Predictably therefore, Spironolactone showed a positive effect on exercise capacity (mean change: VO_2_ peak +1.8 mL/Kg/min, Watt peak +17) and lung DLCO (mean change: +10% of predicted) after six months of treatment [22]. This trend toward significance, though limited for VO2 peak/kg, was first reported by Cicoira *et al*. [23], who observed that the positive effect was even more pronounced at higher doses of spironolactone (e.g., 50 mg).

Despite these benefits, traditional therapies show some limitations. The magnitude of improvement in exercise capacity and ventilatory efficiency remains modest, and a substantial proportion of patients continue to exhibit impaired functional status. Consequently, there is a growing concern in research for adjunctive or alternative pharmacological strategies that can more robustly enhance physical performance and quality of life.

## 4. Angiotensin Receptor–Neprilysin Inhibitors

Angiotensin Receptor–Neprilysin Inhibitors (ARNI) are recommended to treat HFrEF to reduce mortality and HF hospitalization. Since the early evidence on sacubitril/valsartan, several studies have evaluated the drug’s effect on patients’ functional capacity. Of note, the results regarding exercise performance are sometimes conflicting [24, 25, 26, 27].

The ACTIVITY-HF and NEPRIExTol-HF study did not result in a significant benefit on VO_2_ peak when compared with enalapril [28, 29].

Conversely, Vitale *et al*. [30] demonstrated that sacubitril/valsartan significantly improved parameters indicating cardiogenic limitation and deconditioning (VO_2_ peak, Oxygen pulse peak, VO_2_ at anaerobic threshold and VO_2_ work slope) after six months of treatment. Interestingly, the mean increase in functional capacity was substantial and counted at 10% of the VO_2_ percentage of predicted value [30]. The ventilatory efficiency indicated with VE/VCO_2_ slope, along with Forced Expiratory Ventilation at 1st second (FEV_1_) and peak ventilation were also improved with sacubitril/valsartan. These favorable CPET changes seemed to be consistent after one year of treatment [31].

The primary reason for the discrepancies in the conclusions of previous studies stems from the heterogeneity of the HFrEF participant population enrolled. It appears that the most significant improvement in cardiopulmonary fitness was observed in the “intermediate sick” population, where the baseline VO_2_ peak is neither too low nor within the likely normal range.

In line with this consideration, it can be hypothesized that the effect of ARNi on CPET follows a tripartite pattern: effective, grey zone, and ineffective, depending on the selected population. Further studies, particularly those focusing on baseline heart failure severity and concomitant medications, will be necessary to clarify these sources of heterogeneity and may help to confirm this hypothesis.

The dose-related effect on functional capacity remains uncertain and it is still debated. The main evidence suggests that higher doses of sacubitril/valsartan lead to a slightly faster improvement in patients with HFrEF [32]. Nevertheless, the CPET parameters began to show a favorable trend even at low doses [25].

The positive prognostic effect of sacubitril/valsartan was demonstrated by the reduction in the Metabolic Exercise Cardiac and Kindley Index (MECKI) score, which was almost halved after just 6 months of treatment [25].

Finally, evidence suggests that sacubitril and valsartan might have a synergic favorable effect on cardiovascular hemodynamics, ameliorating the conduit vessel function, afterload by limiting blood pressure and diastolic function by reducing left ventricle filling pressure [33]. However, the therapeutic effect extends beyond hemodynamic parameters, pleiotropic effect of reverse remodeling and the reduction of natriuretic peptides or troponin I [34]. Improvements in autonomic function and ventilation are also observed, as demonstrated by enhanced heart rate recovery (HRR) and a reduction in exercise oscillatory ventilation (EOV) prevalence [35].

## 5. Sodium-Glucose Cotransporter-2 Inhibitors

SGLT2i were originally developed as glucose-lowering agents for type 2 diabetes mellitus (T2DM), acting by inhibiting glucose reabsorption in the proximal renal tubules, thereby promoting glucosuria [36]. Beyond glycemic control, SGLT2i exert multiple pleiotropic effects pertinent to HF pathophysiology, including natriuresis, osmotic diuresis, reduction in blood pressure, and weight loss [37]. Mechanistically, they enhance myocardial energy metabolism through a shift toward ketone utilization, promote erythropoiesis, and attenuate inflammation and oxidative stress [38, 39]. In HF patients, these effects converge to improve cardiac remodeling, mitochondrial efficiency, and tissue oxygenation. Furthermore, SGLT2i modulate ferrokinetics, decreasing hepcidin and ferritin levels while increasing soluble transferrin receptor levels. This raises iron availability and potentially augments exercise performance​ [39].

Growing evidence supports the efficacy of SGLT2i in enhancing CPET parameters in HF populations. In the DAPA-VO_2_ trial, Dapagliflozin, significantly increased VO_2_ peak by 1.09 mL/Kg/min at 1 month in patients with stable HFrEF compared to placebo (baseline mean VO_2_ peak = 13.2 ± 3.5 mL/Kg/min) [17]. This improvement, although modest, reached statistical significance. It is noteworthy that in this trial, the effect of Dapagliflozin on VO_2_ peak was on top of appropriate background HF therapy. A post hoc analysis of the trial, which included 76 of the 90 patients enrolled, revealed that these benefits were more pronounced in patients with iron deficiency, highlighting a possible interaction between iron metabolism and SGLT2i-induced functional improvement [39]​.

Meta-analytic data reinforce these findings. A 2023 systematic review and meta-analysis of six studies reported that SGLT2i increased VO_2_ peak by a weighted mean difference (WMD) of 2.02 mL/Kg/min (95% CI: 0.68–3.37; *p* = 0.03) in HF and T2DM populations [40]. Similarly, another meta-analysis of 17 randomized trials with 23,523 HF patients, found a significant increase in VO_2_ peak (mean difference, 1.61 mL/Kg/min; 95% CI: 0.59–2.63; *p* = 0.002) and in 6-minute walk distance (mean difference, 13.09 m; 95% CI: 1.20–24.97 m; *p* = 0.03), supporting a meaningful functional benefit across HF phenotypes [41]​. Gao *et al*. [41] did not find any difference in effect among the various SGLT2 inhibitors evaluated (Empagliflozin, Dapagliflozin, and Canagliflozin), thus suggesting a potential class effect.

Notably, not all studies are uniformly positive. In a prospective real-world cohort, Mapelli *et al*. [42] found no significant change in VO_2_ peak after 6 months of Dapagliflozin therapy in HFrEF patients (median VO_2_ peak 16.2 vs. 16.0 mL/Kg/min; *p* = 0.297), despite improvements in NYHA class (*p* = 0.002), hemoglobin levels (from 13.8 to 14.6 g/dL; *p* < 0.001), and ventilatory efficiency as measured by VE/VCO_2_ slope (from 34.2 to 33.7; *p* = 0.006)​.

Most of the patients enrolled were non-diabetic and NYHA class II at baseline. The Kansas City Cardiomyopathy Questionnaire (KCCQ) demonstrated a mild degree of quality of life (QoL) impairment; similarly, VO_2_ peak and NT-proBNP values indicate a non-severe spectrum of HF. Importantly, most patients were already on optimal background HF therapy, with 81% receiving sacubitril/valsartan, substantially more than in registrative trials such as DAPA-HF [43], possibly explaining the neutral findings on VO_2_ peak [42]. However, to date, no study has evaluated differences in cardiorespiratory impact based on interactions with other HF medications, nor the response to the same drug when administered at doses different from those recommended for HF by current guidelines. Nevertheless, the observed improvements in VE/VCO_2_ and Hb translated into a statistically significant reduction in MECKI score (from 3.3% to 2.8%), suggesting improved 2-year prognosis. These benefits likely reflect an effect of SGLT2i on key determinants of oxygen delivery and utilization according to Fick’s principle, even in the absence of measurable gains in peak VO_2_ [42].

Beyond enhancing exercise capacity, SGLT2i confer important adjunctive benefits in HF management. Their diuretic-like action reduces interstitial and intravascular volume, alleviating pulmonary and systemic congestion, a key determinant of exercise intolerance [44]. Furthermore, they increase hemoglobin levels, likely via enhanced erythropoiesis, improving oxygen delivery during exertion​ [45, 46].

## 6. Glucagon-Like Peptide-1 Receptors Agonists

Glucagon-like Peptide-1 Receptors Agonists (GLP-1 RA), as supported by recent evidence, gained attraction in HFpEF, regardless of diabetes status, especially in the presence of obesity [47]. Sadly, most of the main metanalysis and systematic reviews available evaluated the drug effect on functional capacity only with 6MWT [48]. However, the increase in 6MWT was substantial because the mean difference was 19 meters (up to 22 meters, 95% CI: 1.6–43.0 according to Zhang *et al*. [49]) [50]. Only few trials implemented the CPET. First of all, a small and outdated randomized, double-blind trial, suggested that GLP1-RA infusion did not alter the functional capacity (both for VO_2_ peak and 6MWT) or cardiac output within the first 48 hours of drug infusion [51]. A single small trial from 2017, conducted with Exenatide in diabetic patients only, demonstrated a neutral effect on VO_2_ peak/Kg (*p*-value: 0.146), VO_2_ kinetic, peak workload and respiratory equivalent ratio (RER) [52]. This finding was also confirmed by a recent review by Ni *et al*. [53]. The almost neutral hemodynamic effect was confirmed by Clarke *et al*. [54] with right heart catheterization in a small cohort of patients with advanced HF, where a slight reduction in mixed venous oxygen saturation (SvO_2_ from 62% to 59%) was observed after 15 minutes of GLP-1 infusion. This was accompanied by an increase in peripheral vascular resistance. For this reason, the authors suggested a possible increase in arteriovenous difference (Da-vO_2_) resulting from the peripheral blood toward metabolically more active tissue, leading to a higher oxygen extraction rate [54]. The peripheral muscle effect of GLP1-RA has been linked to mitochondrial improvement in animal models [55]. The currently available literature is limited and will need to be expanded to clarify the pathophysiological mechanisms underle the cardiorespiratory effects of this class of drugs, particularly in patients with HFpEF and when used in combination with SGLT2i.

## 7. Emerging Therapies

Beyond the four pillars, studies investigating the effect of new drugs for HF on functional capacity and cardiopulmonary fitness are lacking. Most clinical trials that validated new drugs chose subjective but widely used parameters for secondary outcomes, such as NYHA and KCCQ. When evaluated, 6MWT primarily represented the sole indirect assessment of functional capacity.

Firstly, vericiguat was approved for worsening HFrEF to reduce mortality and recurrence of HF hospitalizations according to 2021 ESC guidelines (IIb–B class of recommendation) [56]. The only prospective observational study was recently published by Zhan *et al*. [57], who demonstrated a significant improvement in VO_2_ peak, Weber class, VO_2_ at the anaerobic threshold, and VE/VCO_2_ slope after six months of drug treatment. The mean increase in VO_2_ consisted of 3 mL/Kg/min for both peak value and AT [57], meanwhile VE/VCO_2_ slope reduced by 2 points compared to standard treatment. Moreover, the Weber class change was independent of standard clinical and instrumental parameters of HF. Randomized controlled trials (RCTs) are needed to confirm these effects on CPET parameters to establish causal efficacy.

After the initial enthusiasm for GALACTIC-HF, omecamtiv mecarbil was not included in the international ESC/AHA guidelines for HF. In addition to the limited benefit noted regarding cardiovascular mortality and other key endpoints, omecamtiv mecarbil did not demonstrate a positive effect on VO_2_ peak, peak workload and VE/VCO_2_ Slope when assessed by CPET after 20 weeks of treatment (mean change: VO_2_ peak –0.24 mL/Kg/min, peak workload –3.8 Watts, VE/VCO_2_ Slope +0.28) [58, 59].

Despite the promising effect of finerenone on cardiovascular death and hospitalizations for HFpEF and HFmrEF, no study explored the benefit of this drug on functional capacity [60, 61]. The prespecified analysis of FINEARTS-HF, reported a slight increase in the KCCQ score (up to 3 points), but no significant difference in NYHA functional class after 12 months of treatment [62].

## 8. Heart Failure With Preserved Function

The impact of pharmacological therapies on CPET outcomes remains largely unexplored in patients with HFpEF, even though the evidence supporting CPET’s diagnostic and prognostic utility in this subgroup is well established [63]. Exercise capacity is frequently impaired in HFpEF, as evidenced by reduced peripheral oxygen extraction and ventilatory inefficiency (VE/VCO_2_ slope), observed in 40% and 39% of cases, respectively [64].

VO_2_ peak demonstrated an independent prognostic role when below the threshold of 17 mL/Kg/min [65]. Similarly, an elevated VE/VCO_2_ slope (greater than 33) was indicative of more severe disease, higher pulmonary vascular resistance (PVR), and independently predicted increased mortality.

Additionally, integrating CPET-derived parameters with simultaneous stress echocardiography (SE) improves prognostic accuracy compared to echocardiography alone [66].

According to the TOPCAT trial, the patients treated with spironolactone showed modest improvements in health-related QoL, with adjusted mean changes in the KCCQ score of +1.54 at 4 months (*p* = 0.002) and +1.86 at 36 months (*p* = 0.02) [67, 68]. However, the Aldo-DHF trial found no significant improvements in VO_2_ peak or 6MWT with Spironolactone when compared to placebo [69].

While the effect of sacubitril/valsartan on exercise capacity has been extensively investigated in patients with HFrEF, less is known for those with an ejection fraction >40%. Indeed, both the PARAGON and PARAGLIDE-HF trials reported data exclusively on NYHA class and KCCQ [70, 71]. The PARALLAX, actually is the only randomized trial that evaluated the effect of sacubitril/valsartan compared to standard medical therapy on various endpoints, including the distance covered in the 6MWT. After 24 weeks, no significant difference in the 6MWT was observed compared to the control group (9 vs 12.7 m; *p*-value: 0.42) [72].

SGLT2 inhibitors modestly yet significantly enhance functional capacity (VO_2_ peak: +1.1–2 mL/Kg/min) and deconditioning (VO_2_ AT: +1.6 mL/Kg/min) in HFpEF patients, although their effect on VE/VCO_2_ slope remains unclear due to inconclusive data through the available trials [73, 74]. Interestingly, these effects were more pronounced in patients without heart failure, regardless of diabetes status.

In the FINEARTS-HF trial, which evaluated finerenone in patients with HFpEF, the functional endpoints were limited to clinical outcomes (HF events, cardiovascular mortality) and subjective measures such as KCCQ score and NYHA class, with no evidence of objective improvement in functional capacity compared to placebo. In contrast to SGLT2 inhibitors, finerenone has demonstrated no documented impact on CPET-derived parameters in HFpEF [61].

Finally, unlike in HFrEF, the available scientific evidence investigating the effect of pharmacological therapies on standard CPET parameters in HFpEF remains largely limited. This highlights the underutilization of CPET despite its potential benefits, advocating for its wider incorporation into the management of patients with HFpEF.

## 9. Future Perspectives

Given the recent publication of trials investigating cardiac myosin inhibitors in hypertrophic cardiomyopathy (HCM), such as Mavacamten and Aficamten, CPET has begun to appear among study endpoints, recognized as an objective and sensitive tool to detect improvement in patients’ functional capacity [75, 76, 77, 78]. This represents a valuable opportunity for reflection and, ideally, a starting point for future HF trials. When assessing the efficacy of a novel drug for HF, endpoints that evaluate improvements in symptoms and quality of life are as critical as hard endpoints, which include mortality, HF-related hospitalizations, and arrhythmic events. Indeed, a gradual shift from outdated, subjective, and poorly standardized endpoints, such as NYHA class, KCCQ score, and the 6MWT, toward VO_2_ peak and VE/VCO_2_ slope derived from CPET should be encouraged.

To reduce variability and improve clinical interpretation, we suggest a dynamic CPET approach: early evaluation (1–3 months) with OUES to detect submaximal changes (e.g., SGLT2i effects), and late assessment (6–12 months) with VO_2_ peak and VE/VCO_2_ slope to capture structural remodeling (e.g., ARNI response).

Given the weak correlation between LVEF and peak oxygen uptake, a purely LVEF-based assessment of pharmacological response is inadequate and should be replaced by a comprehensive evaluation including clinical (e.g., MECKI score), biomarker, and echocardiographic data.

## 10. Limitations

There are some limitations of this study that have to be acknowledged.

Firstly, finerenone and GLP1-RAs lack specific studies on CPET, so there is still a lot of work to be done to understand their effect on exercise capacity. Similarly, HFpEF has too few studies to generate valid considerations.

Secondly, the study populations were limited and heterogeneous based on age, sex distribution, HF etiology, LVEF subgroup and baseline therapy. There are no placebo-controlled studies of drug combination therapy. As a result, it becomes challenging to identify which patients are likely to respond or not in terms of functional capacity. Such a comparison between pharmacological classes, though of interest, lies beyond the scope of the present work and would necessitate a systematic review or meta-analysis.

Thirdly, CPET remains underutilized in current clinical practice, primarily due to practical barriers such as limited access to the necessary equipment across laboratories and the complexity of result interpretation, which requires specialized training and expertise. The broader implementation of standardized, guideline-directed protocols could offer a potential solution to overcome these challenges.

Finally, this review does not include unconventional and non-pharmacological treatments for HF, such as levosimendan and LVAD support as they warrant a separate, dedicated analysis [79, 80, 81].

Despite these limitations, this is the first review to comprehensively examine the impact of novel heart failure therapies on cardiopulmonary fitness.

## 11. Conclusion

The diagnostic and prognostic role of CPET in HF is well established. Indeed, its use is increasingly widespread among centers specializing in HF management. Evaluating the response to both pharmacological and non-pharmacological treatments has emerged as a key indication for using CPET.

Table 1 summarizes the main effects of new drugs for HF treatment on CPET.

**Table 1.** **Summary of the main effects on CPET of new drugs for heart failure treatment**.

Novel drug treatment	Effect on cardiopulmonary fitness	Time to effect	Number of studies	Study publication period
ARNI	VO_2_ peak/Kg: +10%	3–6 months, Stable effect after 1 year	13	2018–2023
	ppVO_2_ AT: +14%			
	Pulse O_2_: +2 mL/min			
	VO_2_WS: +1			
	VE/VCO_2_ S: –3			
	VE peak: +13 L/min			
	↑ HRR +6 bpm			
	↓ EOV –67%			
	MECKI Score: –2%			
	6MWT unchanged with LVEF >40%			
SGLT2i	VO_2_ peak/Kg: +1.6–2 mL/Kg/min	1–6 months	6	2022–2025
	VE/VCO_2_ S: –0.5			
	6MWT: +13 m			
	MECKI score: –0.5%			
	No CPET studies for HFpEF			
GLP1-RA	No recent CPET study	>48 h	5	2010–2025
	Neutral effect on VO_2_ peak	Within 6 months		
	6MWT: +19 meters			
	Neutral hemodynamic effect			
	↑ D(a-v) O_2_			
Vericiguat	VO_2_ peak/Kg: +3 mL/Kg/min	6 months	1	2025
	VO_2_ AT: +2 mL/Kg/min			
	VE/VCO_2_ S: –2			
Finerenone	No specific CPET study	12 months	NA	2024
	Increase KCCQ (+3 pts)			
	NYHA unchanged			
Omecamtiv mecarbil	Not significant	20 weeks	1	2022

ARNI, Angiotensin Receptor–Neprilysin Inhibitors; SGLT2i, Sodium-Glucose Cotransporter-2 Inhibitors; GLP1-RA, Glucagon-Like Peptide-1 Receptor Agonists; VO_2_, Oxygen Uptake; ppVO_2_, Percentage of Predicted VO_2_ Peak; VO_2_ WS, VO_2_ work slope; VE/VCO_2_ S, Relationship between Ventilation and Carbom Dioxide Production; AT, Anaerobic Threshold; DLCO, Diffusing Capacity of the Lung for Carbon Monoxide; PAP, Pulmonary Artery Hypertension; 6MWT, Six Minute Walking Test; EOV, Exercise Oscillatory Ventilation; HRR, Heart Rate Recovery; LVEF, Left Ventricular Ejection Fraction; NA, Not Available; CPET, Cardiopulmonary Exercise Testing; KCCQ, Kansas City Cardiomyopathy Questionnaire; NYHA, New York Heart Association. ↑, means increase. ↓, means decrease.

As represented, the main number of studies focused on sacubitril/valsartan. Assessing the interval at which CPET was repeated reveals significant heterogeneity, ranging from 6 months to 1 year. Despite growing scientific evidence supporting the repetition of CPET in HF patients, this finding highlights the lack of a clearly defined optimal timeframe for CPET repetition [82, 83, 84]. Previous studies discourage repeating CPET before six months of follow-up. Therefore, the most reasonable solution aims at a tailored timing of repetition according to the patient’s risk profile and the pathophysiological mechanism of drug action.

Finally, CPET provides a holistic assessment of the body’s “engine”, allowing for the detection of global improvements in patients with heart failure even when these changes are subclinical and would otherwise go unnoticed during a standard clinical evaluation. Therefore, broader use of CPET in this context is highly desirable, especially considering that surrogate measures of functional capacity, such as the 6MWT [85, 86], offer significantly less information and often represent maximal effort in many patients, particularly those with more advanced disease.

## Abbreviations

6MWT, Six Minute Walking Test; ACEi, Angiotensin-Converting Enzyme Inhibitors; ARBs, Angiotensin II Receptor Blockers; ARNI, Angiotensin Receptor–Neprilysin Inhibitors; BB, Beta blockers; CPET, Cardiopulmonary Exercise Testing; DLCO, Diffusing Capacity of the Lung for Carbon Monoxide; FEV1, Forced Expiratory Volume in One Second; GLP-1 RA, Glucagon-Like Peptide-1 Receptor Agonists; HF, Heart Failure; HFpEF, Heart Failure with Preserved Ejection Fraction; HFrEF, Heart Failure with Reduced Ejection Fraction; HR, Heart Rate; HRR, Heart Rate Recovery; KCCQ, Kansas City Cardiomyopathy Questionnaire; MRAs, Mineralocorticoid Receptor Antagonists; SV, stroke volume; VE/VCO_2_ slope, Relationship between Ventilation and Carbon Dioxide production; VO_2_, Oxygen Uptake; QoL, Quality of Life; NT-pro-BNP, N-terminal pro Brain Natriuretic Peptide.
